# P-1761. Safety and Efficacy of Antimicrobial Optimization based on Negative Results from BioFire FilmArray Pneumonia Panel

**DOI:** 10.1093/ofid/ofae631.1924

**Published:** 2025-01-29

**Authors:** Noah Yoo, Xian Jie Cheng, Juri Chung, Shalinee Chawla, Ioannis Zacharioudakis, Yanina Dubrovskaya

**Affiliations:** NYU Langone Health - Long Island, Mineola, New York; Northwell Health, Huntington, New York; NYU Langone Health - Long Island, Mineola, New York; NYU Langone Health - Long Island, Mineola, New York; New York University, New York city, New York; NYU Langone Health, New York, New York

## Abstract

**Background:**

The BioFire FilmArray Pneumonia (BFP) panel is a multiplexed nucleic acid test intended for detection of multiple respiratory viral and bacterial specimens from sputum or bronchoalveolar lavage (BAL) specimens, with high specificity and turnaround time. Efficacy and safety of de-escalation strategies in patients with negative BFP have not been adequately described.

**Figure:**
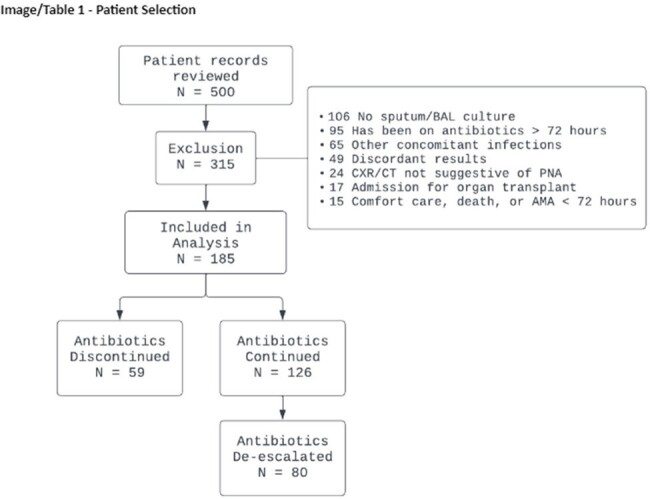

**Methods:**

This was a multi-center, retrospective analysis of adult patients with suspected pneumonia and sputum or BAL samples that tested negative on BFP. Patients for whom antibiotic therapy was discontinued (ATDC Group) were compared to patients who had their antibiotic therapy continued (ATC Group). Outcomes included in-hospital mortality, 30-day readmission, and hospital and ICU length of stay. Impact on the total number of days on antibiotics and the safety of the intervention were also assessed.

**Figure:**
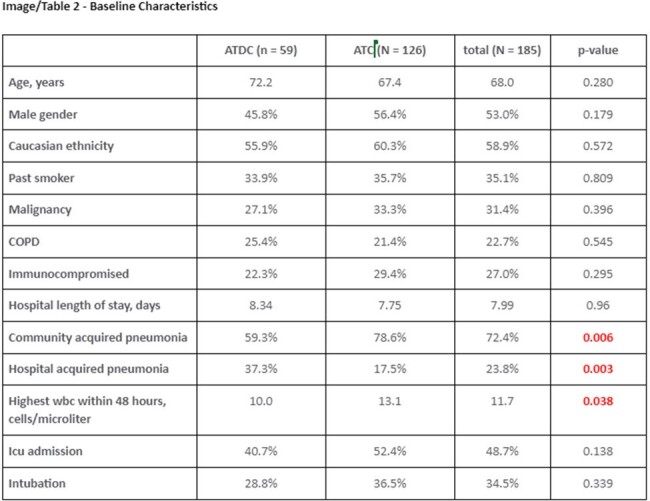

**Results:**

Out of 500 patients with negative BFP assay, a total of 185 patients were included in the final analysis (59 ATDC Group vs. 126 ATC Group). 67.6% of samples were sputum and 32.4% were BAL in the entire cohort. The groups were generally well-balanced in baseline characteristics, but more patients in ATC Group had community-acquired pneumonia (59% vs 79%, p = 0.006), higher initial white blood cell count (10 vs 13, p = 0.038), and higher quick Pitt Bacteremia Score (1 [IQR 0-1] vs 1 [IQR 0-3], p = 0.020). The ATDC Group had significantly shorter days of total antibiotic therapy (1.0 days vs 10.6 days, p < 0.001). 30-day readmission rates (16.95% vs 15.87%, p = 0.853) and the hospital length of stay (8.34 days vs 7.75, p = 0.960) did not differ. The ATDC Group experienced fewer AKI (8% vs 37%, p = 0.004).

Duration of Antibiotics

**Figure:**
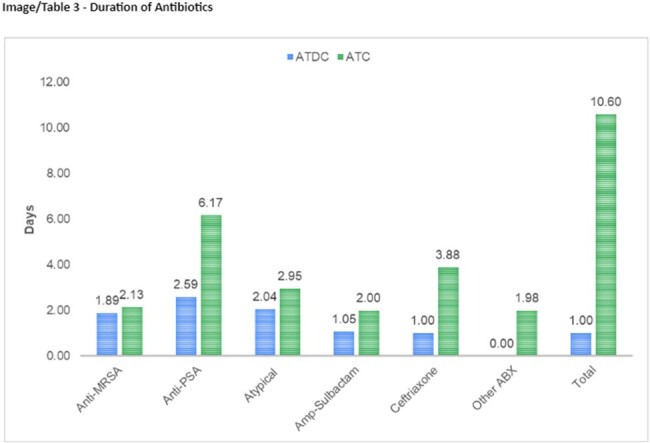

**Conclusion:**

Antibiotic discontinuation based on BFP was associated with significant reduction in the number of total days on antibiotics. Discontinuation was not associated with increases in in-hospital mortality, length of stay, or readmission, but was associated with a significant reduction in acute kidney injury.

**Figure:**
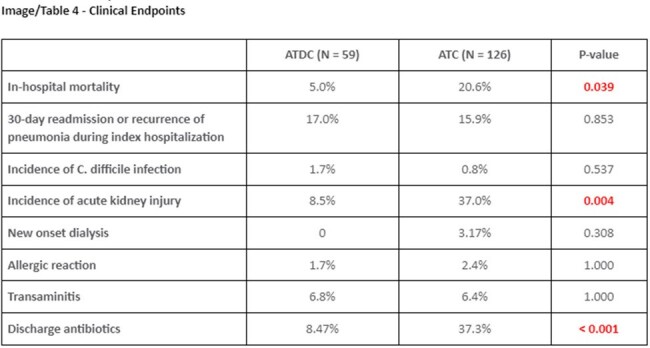

**Disclosures:**

**All Authors**: No reported disclosures

